# Unlocking the Green Gold: Exploring the Cancer Treatment and the Other Therapeutic Potential of Fucoxanthin Derivatives from Microalgae

**DOI:** 10.3390/ph17070960

**Published:** 2024-07-18

**Authors:** Fatouma Mohamed Abdoul-Latif, Ayoub Ainane, Ibrahim Houmed Aboubaker, Ali Merito Ali, Houda Mohamed, Pannaga Pavan Jutur, Tarik Ainane

**Affiliations:** 1 Center for Research and Study of Djibouti, Medicinal Research Institute, Djibouti City P.O. Box 486, Djibouti; 2 Superior School of Technology, University of Sultan Moulay Slimane, P.O. Box 170, Khenifra 54000, Morocco; 3 Peltier Hospital of Djibouti, Djibouti City P.O. Box 2123, Djibouti; 4 Omics of Algae Group, Industrial Biotechnology, International Centre for Genetic Engineering and Biotechnology, Aruna Asaf Ali Marg, New Delhi 110067, India; pavan.jutur@icgeb.org

**Keywords:** anti-cancer, biochemistry, fucoxanthin, prospects, therapeutic, microalgae

## Abstract

Fucoxanthin, a carotenoid widely studied in marine microalgae, is at the heart of scientific research because of its promising bioactive properties for human health. Its unique chemical structure and specific biosynthesis, characterized by complex enzymatic conversion in marine organisms, have been examined in depth in this review. The antioxidant, anti-inflammatory, and anti-cancer activities of fucoxanthin have been rigorously supported by data from in vitro and in vivo experiments and early clinical trials. Additionally, this review explores emerging strategies to optimize the stability and efficacy of fucoxanthin, aiming to increase its solubility and bioavailability to enhance its therapeutic applications. However, despite these potential benefits, challenges persist, such as limited bioavailability and technological obstacles hindering its large-scale production. The medical exploitation of fucoxanthin thus requires an innovative approach and continuous optimization to overcome these barriers. Although further research is needed to refine its clinical use, fucoxanthin offers promising potential in the development of natural therapies aimed at improving human health. By integrating knowledge about its biosynthesis, mechanisms of action, and potential beneficial effects, future studies could open new perspectives in the treatment of cancer and other chronic diseases.

## 1. Introduction

Tiny microorganisms known as microalgae, often unnoticed by the naked eye, have garnered significant interest from researchers worldwide in recent decades [1]. Their appeal stems from their remarkable potential in medicine and sustainable biotechnology, aligning perfectly with the global pursuit of healthy lifestyles and sustainable development [2]. These microscopic organisms in seawater or freshwater play an important role in marine ecosystems and offer numerous opportunities to satisfy the increasing demand for natural resource-based products [3]. One of the most fascinating aspects of microalgae is their biological diversity, as they are rich in bioactive compounds of various secondary metabolites, including polysaccharides, lipids, proteins, enzymes, vitamins, and carotenoids [4]. These substances have diverse applications across multiple sectors, such as medicine, pharmaceuticals, nutraceuticals, cosmetics, and energy. Extensive research has validated the potential of microalgae as a reliable and environmentally friendly raw material for producing valuable resources, paving the way for new advancements in biotechnology [4].

Yellow to orange-red terpenoid pigments, called carotenoids, are synthesized by specific organisms, particularly microalgae. These substances can act as antioxidants by neutralizing free radicals [5]. Carotenoids are divided into two categories: xanthophylls, which contain oxygen, and carotenes, which are pure hydrocarbons devoid of oxygen. Some xanthophylls produced by higher plants, such as violaxanthin, antheraxanthin, zeaxanthin, neoxanthin, and lutein, can also be synthesized by green microalgae [6]. However, these have additional xanthophylls such as loroxanthin, astaxanthin, and canthaxanthin [7]. Diatom microalgae can produce diatoxanthin, diadinoxanthin, and fucoxanthin [8]. Microalgae such as *Haematococcus pluvialis*, *Dunaliella salina*, *Blakesleatrispora* sp., *Desmodesmus* sp., *Euglena gracilis*, *Tisochrysis lutea*, *Isochrysis galbana*, *Phaeodactylum tricornutum*, and *Cylindrotheca closterium* are among the most studied due to their ability to produce carotenoids in significant quantities [9,10]. These microorganisms synthesize a range of rare carotenoids, opening the way to innovative industrial and scientific applications. Carotenoids, especially fucoxanthin, stand out for their powerful antioxidant and anti-inflammatory properties, which benefit human health by helping to combat various chronic diseases [11].

Fucoxanthin, a marine carotenoid found in the photosynthetic cells of diatoms, is gaining significant interest due to its numerous health benefits [12]. Research has highlighted its effectiveness in weight management, cardiovascular health, and blood sugar regulation, and its promising potential in cancer prevention [13]. From a biotechnology perspective, fucoxanthin is extensively used in producing dietary supplements, nutraceuticals, and cosmetics, underscoring its vital role in the health and wellness industry [14]. Beyond medical applications, fucoxanthin shows promise in sustainable production and the development of value-added by-products [15]. The cultivation of microalgae for the extraction of fucoxanthin offers a promising prospect for eco-compatible production, circumventing the environmental challenges frequently linked to the scale-up of this molecule.

This comprehensive review also examines fucoxanthin derivatives from microalgae and their anticancer and other medical implications. It delves into fucoxanthin’s complex chemistry and biochemistry and its essential role in enhancing human health. Additionally, it addresses future challenges and potential prospects for using and further developing fucoxanthin and its derivatives in various applications.

## 2. Chemistry of Fucoxanthin and Its Derived Compounds

**Figure 1:**
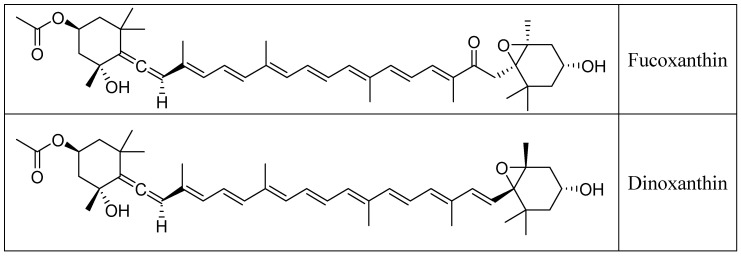

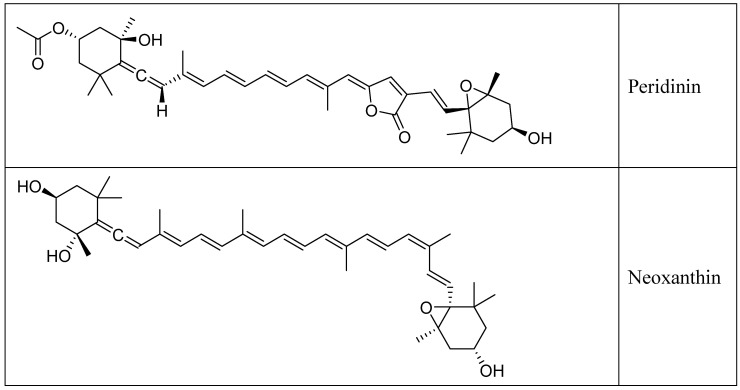
Fucoxanthin, Dinoxanthin, Peridinin, and Neoxanthin structures.

**Figure 2:**
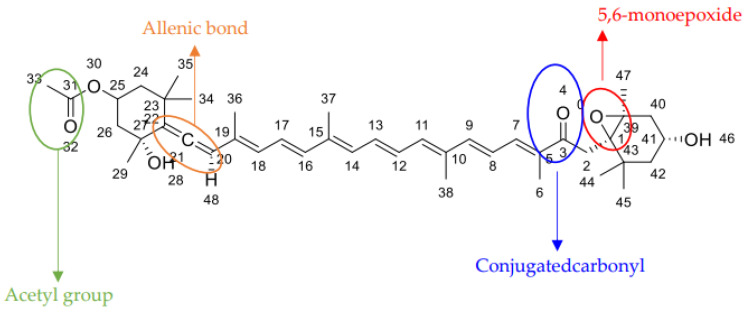
Key characterized groups and bands of Fucoxanthin.

Fucoxanthin, a distinctive xanthophyll with the chemical formula C_42_H_58_O_6_, is an essential secondary metabolite in the chloroplasts of various aquatic species, including microalgae, giving them brown or olive green colors [16]. Its molecular structure, close to dinoxanthin, peridinin, and neoxanthin (Figure 1), is distinguished by an unusual allenic bond, nine conjugated double bonds, and an epoxide at position 5,6, as well as several oxygenated functional groups, including alcohols, epoxides, ketones, and esters (Figure 2) [17].

The intricate chemical composition and molecular conformation of fucoxanthin significantly influence its physicochemical and biological properties. Its effectiveness in various biological processes, including its biochemical mechanisms in living organisms, is critically dependent on storage conditions and the formation of other isomers, which is essential for its potential medical and pharmaceutical applications [18].

Like other carotenoids, fucoxanthin is prone to degradation during storage due to temperature, light, oxygen, enzymes, unsaturated lipids, and other pro-oxidant agents. Isomerization, a common occurrence, can lead to the formation of cis isomers influenced by processing conditions, environment, and the specific type of carotenoid [19]. During the purification process of fucoxanthin, three prominent peaks are typically observed, corresponding to the transform and two cis isomers, with an increased proportion of cis isomers at higher extraction temperatures. The sensitivity of fucoxanthin to heat results in the degradation of its total form and the all-trans form when heated between 25 and 100 °C. High temperatures also promote the formation of the cis-13 and cis-15 isomers and degrade the cis-9 isomer. The extraction of fucoxanthin from Undaria pinnatifida algae by an ultrasonic process using rapeseed oil as a solvent made it possible to obtain an oil rich in fucoxanthin. High internal phase emulsions (HIPEs), stabilized by 2% gelatin (GE)-based microgels, exhibit a small, uniform particle size and a stable microstructure. These HIPEs show excellent viscoelasticity, high recovery rate, and thixotropy, thus optimizing their performance for 3D food printing. HIPEs protect fucoxanthin from chemical degradation and improve its bioaccessibility by promoting the release of free fatty acids (FFA) during simulated in vitro digestion. The use of rapeseed oil as an extraction solvent offers a green and stable alternative to traditional organic solvents, showing strong potential for the future design of oil-soluble bioactives and flavors [20].

**Figure 3:**
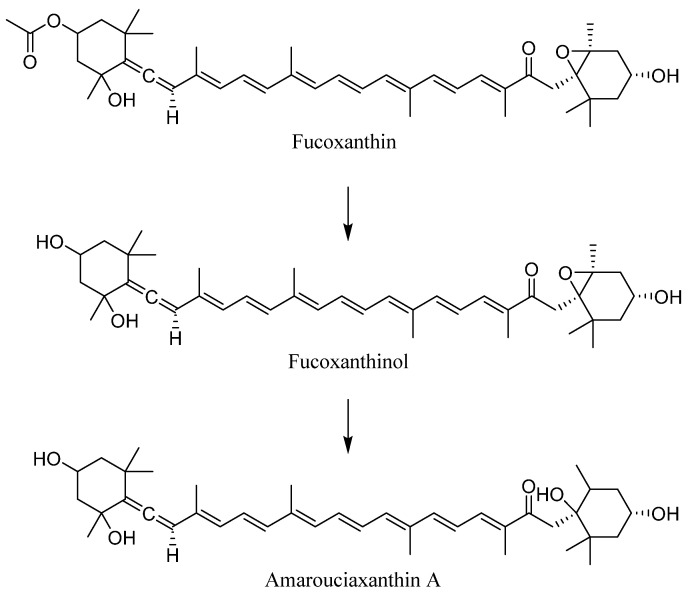
The chemical structures of the two fucoxanthin derivatives: fucoxanthinol and amarouciaxanthin A.

The metabolic transformation of fucoxanthin produces two major derivatives: fucoxanthinol and amarouciaxanthin A (Figure 3) [21]. Fucoxanthinol, the major metabolite generated by hydrolysis in the gastrointestinal tract shortly after ingestion, plays an essential role in the biological effects of fucoxanthin. Conversely, amarouciaxanthin A comes mainly from fucoxanthinol present in the liver. Studies in mice have shown that the conversion of fucoxanthinol to amarouciaxanthin A occurs primarily in liver microsomes and requires NAD(P)^+^ as a cofactor. Additionally, fucoxanthinol added to culture medium via HepG2 cells has also been shown to convert to amarouciaxanthin A, indicating that this metabolic pathway is active in cultured human cells [22].

## 3. Microalgae as a Source of Fucoxanthin

Microalgae, although often unknown to the general public, captivate researchers with their distinctive properties and exceptional diversity. These microscopic organisms, usually smaller than 50 μm, play a vital role in the stability of aquatic ecosystems as photosynthetic organisms. Their ability to transform sunlight, carbon dioxide, and water into organic compounds makes them essential primary producers, contributing to biomass and nutrient cycling [23].

**Figure 4:**
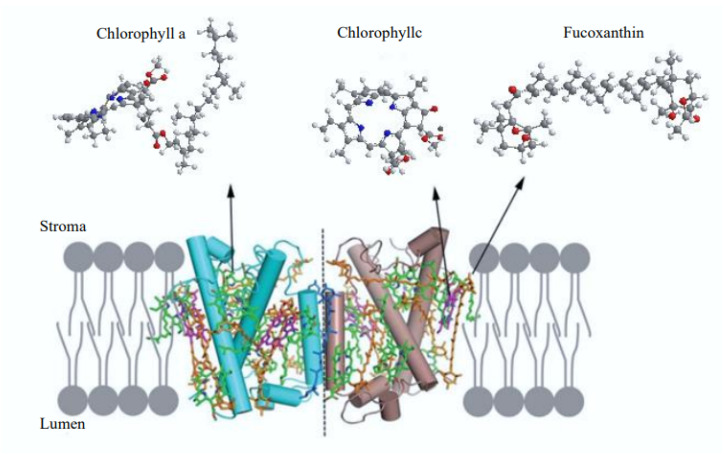
Model of the structure of fucoxanthin-chlorophyll proteins (FCP) in thylakoid membranes.

Microalgae are notable for their ability to synthesize diverse bioactive compounds, including carotenoids, which are essential for photosynthesis and beneficial to human health due to their antioxidant properties [24]. Among these carotenoids, fucoxanthin is particularly valuable in the medical and pharmaceutical fields. Found in the chloroplasts of various microalgae species such as *Isochrysis* sp. and *Tisochrysis* sp., fucoxanthin is a xanthophyll that accumulates in the cytosolic lipid droplets of marine diatoms like *Phaeodactylum tricornutum* [25,26]. As an antenna pigment in light-harvesting complexes, fucoxanthin efficiently transfers energy to the photosynthetic electron transport chain, absorbing light with high efficiency and transferring it to chlorophyll a (>80%). This energy transfer is primarily due to a spectral overlap between the donor and acceptor components of the complex [27]. Additionally, the short excited-state lifetime of fucoxanthin facilitates rapid energy transfer, as indicated by low fluorescence. Fucoxanthin also plays a role in protecting against excessive light and has potent antioxidant activity. Fucoxanthin chlorophyll proteins, complexes that combine fucoxanthin, chlorophyll a, and chlorophyll c with proteins, are the main light-harvesting complexes in microalgae. While similar complexes are present in brown algae, they adapt differently to high light intensities through various non-photochemical quenching mechanisms. In macroalgae, fucoxanthin chlorophyll proteins include violaxanthin and zeaxanthin via the violaxanthin cycle, whereas in diatom microalgae, they bind to diadinoxanthin and diatoxanthin through the diadinoxanthin-diatoxanthin cycle for photoprotection (Figure 4) [28].

Recent studies have documented varying concentrations of fucoxanthin in different microalgae strains, highlighting these organisms’ diversity and biotechnological potential (Table 1). Ishika et al. (2017) [29] reported 1.2 mg/g fucoxanthin in *Amphora* sp., while *Chaetoceros muelleri* presented 2.92 mg/g. Tokushima et al. (2016) [30] recorded a 12.0 mg/g concentration in *Cyclotella cryptica*, highlighting its capacity to store this pigment. Similarly, Ryabushko et al. (2017) [31] observed a concentration of 15.2 mg/g in *Cylindrotheca closterium*, while Wang et al. (2018) [32] noted levels varying from 15.2 to 25.5 mg/g among different strains of this species.

Popovich et al. (2020) [33] reported an exceptionally high concentration of 38 mg/g of fucoxanthin in *Halamphora coffeaeformis*, underscoring its potential as a valuable source of this carotenoid. This intra-species diversity emphasizes the importance of carefully selecting microalgae strains to optimize fucoxanthin production for biotechnological applications. Research by Wu et al. (2016) [34] on *Phaeodactylum tricornutum* also demonstrated significant variability in fucoxanthin concentrations, ranging from 5.5 mg/g to a broad range of 9.9 to 59.2 mg/g, as noted by McClure et al. (2018) [35], again highlighting the intra-species diversity of this carotenoid. Studies on other species, such as *Isochrysis zhangjiangensis* by Li et al. (2019) [36] and *Odontella aurita* by Xia et al. (2013) [37], confirmed their potential as rich sources of fucoxanthin, with concentrations of 23.3 mg/g and 18.47 mg/g, respectively. Mohamadnia et al. (2021) [38] optimized the culture medium for *Tisochrysis lutea*, achieving a remarkable fucoxanthin concentration of 79.40 mg/g. This significant advancement demonstrates the effectiveness of optimizing culture conditions to enhance the production of this carotenoid.

**Table 1:** Variability of fucoxanthin concentrations in the main strains of microalgae.

Microalgae Species	Fucoxanthin (mg/g)	Reference
*Amphora* sp.	1.2	[29]
*Chaetoceros muelleri*	2.9	[29]
*Phaeodactylum tricornutum*	5.5	[34]
*Cyclotella cryptica*	12.0	[30]
*Odontella aurita*	18.5	[37]
*Isochrysis zhangjiangensis*	23.3	[36]
*Cylindrotheca closterium*	25.5	[32]
*Halamphora coffeaeformis*	38.0	[33]
*Phaeodactylum tricornutum*	59.2	[35]
*Tisochrysis lutea*	79.4	[38]

## 4. Biosynthesis of Fucoxanthin

**Figure 5:**
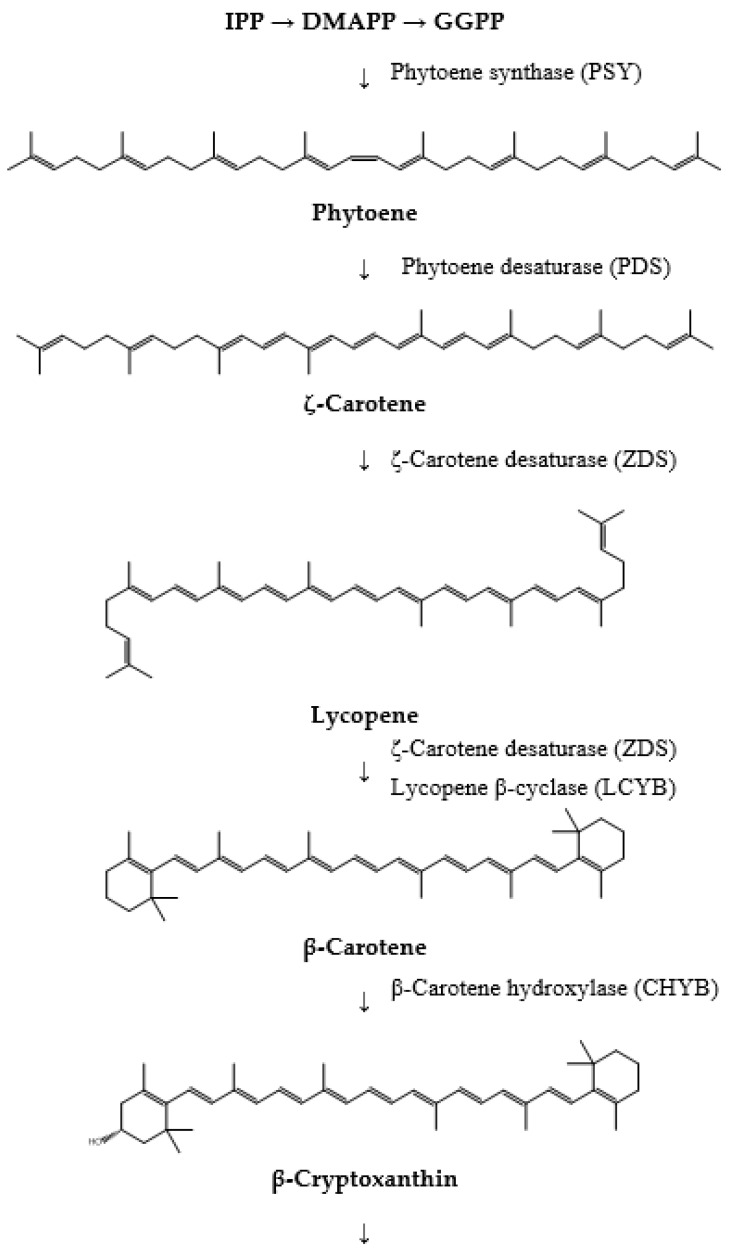

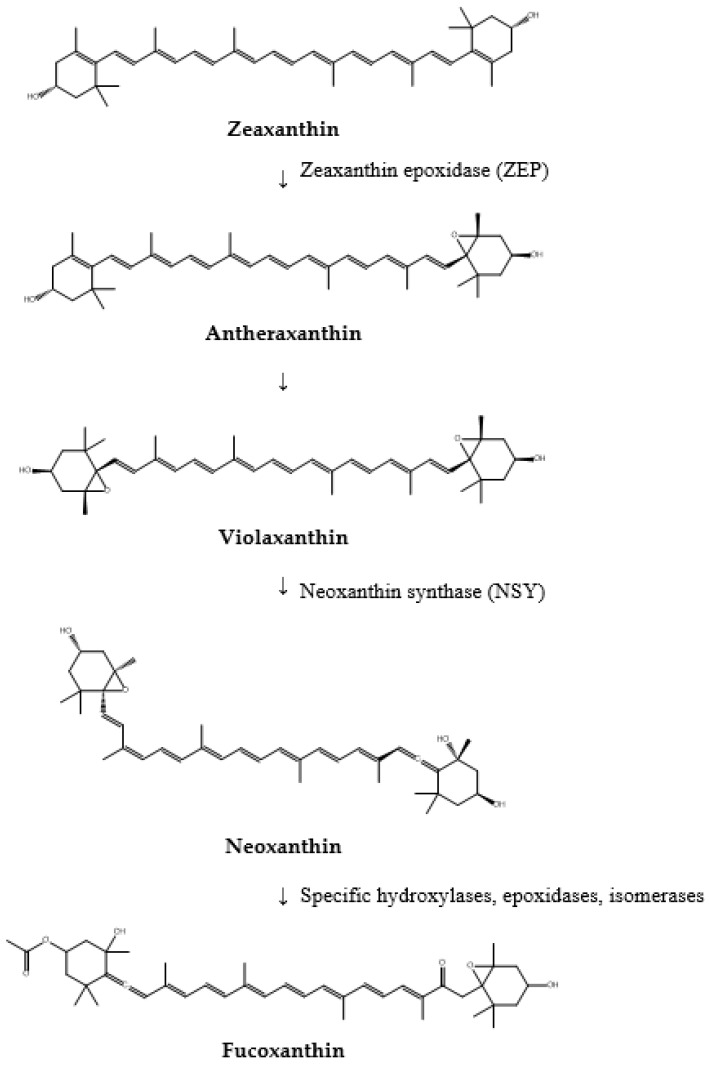
Main steps of the fucoxanthin biosynthesis pathway in the majority of microalgae species.

Fucoxanthin biosynthesis in microalgae is a complex and multifactorial mechanism involving a series of enzymatic reactions [39]. This process begins with forming carotenoid precursors such as β-carotene and producing fucoxanthin. Each step in this metabolic pathway is catalyzed by specific enzymes that add, modify, or rearrange functional groups to form the distinct structure of fucoxanthin [40,41,42,43,44,45]. The main stages of the fucoxanthin formation mechanism are described below (Figure 5):

1. The initial step in fucoxanthin biosynthesis involves the production of the C5 building blocks, isopentenyl pyrophosphate (IPP) or its isomer, dimethylallyl diphosphate (DMAPP). These molecules can be synthesized from acetyl-CoA through the cytosolic mevalonic acid (MVA) pathway or from pyruvate and glyceraldehyde-3-phosphate (G3P) via the plastid methylerythritol 4-phosphate pathway (MEP). Although both pathways yield the same end product, carotenoid synthesis predominantly utilizes IPP or DMAPP derived from the MEP pathway.
2. The formation of phytoene marks the initial specific step in the carotenoid pathway. This process is catalyzed by phytoene synthase (PSY), which facilitates the head-to-head condensation of two geranylgeranyl diphosphate (GGPP) molecules to produce phytoene. Phytoene is pivotal as it serves as the first distinct intermediate in this biosynthetic pathway.
3. The conversion of phytoene into lycopene follows next and involves several enzymes. Phytoene desaturase (PDS), ζ-carotene desaturase (ZDS), and carotene isomerase (CRTISO) sequentially desaturate phytoene to generate ζ-carotene, subsequently leading to the formation of lycopene. Lycopene represents the initial colored carotenoid produced in this biosynthesis pathway.
4. Subsequently, lycopene undergoes cyclization to form β-carotene, a process catalyzed by lycopene β-cyclase (LCYB). β-carotene is an important precursor in fucoxanthin biosynthesis, marking a significant step in converting linear carotenoids into cyclic compounds.
5. β-carotene is then hydroxylated to produce zeaxanthin. This biosynthetic step involves β-carotene hydroxylase (CHYB), which introduces hydroxyl groups to β-carotene, forming β-cryptoxanthin and subsequently zeaxanthin.
6. Zeaxanthin undergoes epoxidation to form antheraxanthin and then violaxanthin. Zeaxanthin epoxidase (ZEP) catalyzes this reaction by adding epoxy groups to zeaxanthin, thereby transforming the carotenoids into more complex and functional forms.
7. Violaxanthin is further converted into neoxanthin by neoxanthin synthase (NSY). This transformation represents another critical step in the biosynthetic pathway, laying the groundwork for subsequent modifications that lead to the formation of fucoxanthin.
8. Finally, neoxanthin undergoes a series of intricate enzymatic modifications to synthesize fucoxanthin. These reactions involve various enzymes, including hydroxylases, epoxidases, and specific isomerases, which introduce hydroxyl and epoxy groups and facilitate structural rearrangements necessary for forming fucoxanthin’s unique allene bond.

## 5. Anticancer Activities of Fucoxanthin

The anticancer properties of fucoxanthin are multiple and diverse (Table 2), impacting various types of neoplasms with specific mechanisms of apoptosis induction and cell cycle regulation [46]. Its selective properties, sparing healthy cells while targeting neoplastic cells, make it particularly promising for developing new oncological therapies.

Glioma is the most common primary tumor of the central nervous system. Fucoxanthin is cytotoxic to human glioma cell lines U87 and U251, with no inhibitory effect on normal neurons. It induces apoptosis of glioblastoma multiforme cells by increasing the levels of pro-apoptotic proteins, including cleaved PARP, caspase-3, and caspase-9, and reducing the accumulation of phosphorylated Akt and mTOR proteins. Control experiments with PI3K inhibitors confirmed that fucoxanthin-induced apoptosis is linked to inhibition of the PI3K/Akt/mTOR pathway [47].

Breast carcinoma is a common malignancy that seriously threatens the physical and mental health of women. Fucoxanthin has anti-angiogenic effects, inhibiting the proliferation, migration, and formation of tubular structures by human lymphatic endothelial cells (HLEC). It also reduces lymphatic microvessel density in breast carcinoma models, highlighting its potential as an anti-lymphangiogenic agent to prevent metastasis. Fucoxanthin and its metabolite fucoxanthinol lessen the viability of MCF-7 and MDA-MB-231 cells in a dose- and time-dependent manner. The combination of fucoxanthin and adriamycin selectively activates oxidative stress-mediated apoptosis of breast carcinoma cells while protecting cardiomyocytes from the toxic effects of doxorubicin [48].

Fucoxanthin inhibits the proliferation of nasopharyngeal carcinoma cells by inducing autophagy. It also prevents the development of non-small cell lung carcinoma at a relatively safe dose for normal cells by inducing apoptosis of neoplastic cells by regulating the expression of p53, p21, Fas, PUMA, Bcl-2, and caspase-3/8. Fucoxanthin induces the nuclear translocation of p53, stimulating its function as a transcriptional activator in cancer cells and reducing the levels of proteins associated with cancer cell proliferation, survival and metastasis [49].

Cervical carcinoma is currently the most common gynecological malignancy. Fucoxanthin significantly increases apoptosis in cervical cancer cell lines HeLa, SiHa, and CaSki in synergy with the TRAIL protein. TRAIL, a member of the TNF family of cytokines, selectively induces apoptosis of tumor cells without affecting normal cells. TRAIL-mediated apoptosis is enhanced by fucoxanthin via the PI3K/Akt/NF-κB pathway, which increases apoptosis of cervical cancer cells [50].

Colorectal carcinoma is a common cancer of the digestive tract. Fucoxanthin significantly reduced the proliferation of human colorectal cancer cell lines Caco-2, HT-29, and DLD-1 by inducing DNA fragmentation. More recently, it has been reported that fucoxanthin can effectively prevent colorectal cancer in a carcinogenic AOM/DSS mouse model. It inhibits colon damage and promotes resistance to colorectal cancer by activating the anoikis pathway and inhibiting tumor growth [51].

Hepatocellular carcinoma is one of the most common and deadly cancers worldwide. Studies have shown that fucoxanthin has a significant therapeutic effect against diethylnitrosamine-induced liver cancer in rats. It restores normal body weight, serum albumin levels, antioxidant enzymes, liver enzymes, serum bilirubin, and stress markers. Fucoxanthin protects hepatocytes by increasing antioxidant enzymes such as superoxide dismutase and catalase and eliminating lipid peroxide metabolites. Another study demonstrated that fucoxanthin-enriched extracts were cytotoxic to HepG2 liver cancer cells in a dose- and time-dependent manner. Fucoxanthin also reduces the viability of HepG2 cells by inhibiting cyclin D expression and inducing cell cycle arrest in the G_0_/G_1_ phase [52].

Chronic myelogenous leukemia (CML) is a malignancy resulting from the clonal proliferation of hematopoietic stem cells. Imatinib, although commonly used, has many adverse effects and cannot eradicate the pathogenic gene. Combined with imatinib and doxorubicin, Fucoxanthin showed cytotoxic effects on human leukemia cell lines K562 and TK6. Fucoxanthin also induces apoptosis of human acute promyelocytic leukemia cells, which is related to the early loss of mitochondrial membrane potential and the activation of caspase-3. It influences HEL human erythroid leukemia cells by reducing their viability, increasing cell apoptosis and causing cell cycle arrest in the G_0_/G_1_ phase. Fucoxanthin opens the mitochondrial membrane permeability transition channel, reducing mitochondrial transmembrane potential and regulating Bcl-2 and Bax gene expression, leading to increased expression of pro-apoptotic caspase-3 [53].

Gastric cancer, with its high morbidity and mortality, represents a significant challenge in oncology. Traditional chemotherapy for this type of cancer lacks specificity and causes substantial damage to normal cells. Fucoxanthin shows promising anticancer activity by reducing Mcl-1 and STAT3 protein expression in SGC-7901 and BGC-823 gastric cancer cells, causing cell cycle arrest in the S phase and apoptosis in the G_2_/M phase. It is not toxic to normal cells, which is essential for therapeutic application. Studies have also revealed that fucoxanthin can induce autophagy and apoptosis by increasing the expression of beclin-1, LC3, and cleaved caspase-3 while decreasing the expression of Bcl-2 [54].

In silico studies on the anticancer properties of fucoxanthin have significantly enriched our bioinformatics understanding of its therapeutic potential against cancer. This cutting-edge research used advanced models such as molecular dynamics simulation, molecular docking, ADME (Absorption-Distribution-Metabolism-Excretion) and QSAR (Quantitative Structure–Activity Relationship) analysis of computational chemistry to explore the mechanisms of action of this promising molecule.

Seminal studies by Januar et al. (2012) [55] extensively investigated fucoxanthin’s cytotoxic properties, employing an in silico approach that revealed its stable complex formation with tubulin at the colchicine binding site. This discovery opened new avenues for targeted cancer therapies centered on microtubule depolymerization and cell cycle arrest. Furthermore, Jung et al. (2017) [56] examined fucoxanthin’s impact on monoamine oxidase-A (MAO-A) and monoamine oxidase-B (MAO-B) through molecular docking studies, demonstrating significant enzyme inhibition. Their findings suggested that fucoxanthin could influence critical metabolic pathways in cancer treatment. Building on these studies, Garg et al. (2019) [57] delved deeper into fucoxanthin’s interaction with the p53-mortalin complex using molecular docking and dynamics simulations. Their research confirmed its ability to disrupt essential interactions between p53 and mortalin, reinforcing its potential as a competitive inhibitor in anticancer therapeutic strategies.

Moreover, Dibha et al. (2022) [58] extended the investigation into fucoxanthin’s potential in breast cancer by focusing on its interactions with nuclear factor kappa B (NF-kB) via molecular docking simulations. Their analysis underscored essential physicochemical and pharmacokinetic characteristics of fucoxanthin essential for its therapeutic development, including challenges such as limited blood–brain barrier penetration and restricted gastrointestinal absorption. Additionally, Padmi et al. (2023) [59] explored fucoxanthin’s potential against melanoma cells by targeting the MC1R receptor, adding a novel dimension to studying its anticancer properties. These collective studies highlight fucoxanthin’s diverse mechanisms and potential applications in cancer therapy, underscored by its interactions with key molecular targets and pathways in cancer progression and metabolism.

## 6. Medical Applications of Fucoxanthin

### 6.1. Antioxidative Activities

Fucoxanthin possesses a distinctive chemical structure characterized by an allenic bond, an epoxide, and a hydroxyl group. These features endow fucoxanthin with potent antioxidant properties by enabling it to interact effectively with various free radicals. The allenic bond’s high reactivity facilitates the neutralization of free radicals, while the epoxide group enhances fucoxanthin’s ability to react with reactive oxygen species (ROS). Moreover, the hydroxyl group enhances fucoxanthin’s solubility in biological fluids, thereby improving its antioxidant efficacy [60].

Oxidative stress arises from an imbalance between ROS production and the body’s antioxidant defense mechanisms. Fucoxanthin mitigates oxidative stress through several mechanisms. It activates antioxidant enzymes such as catalase, superoxide dismutase (SOD), and glutathione peroxidase (GSH-Px), important for ROS breakdown. Additionally, fucoxanthin modulates antioxidant signaling pathways by stimulating Nrf2 (nuclear factor erythroid 2–related factor 2), which enhances the expression of genes encoding antioxidant enzymes like NAD(P)H quinone oxidoreductase 1 (NQO1) [61].

In vitro studies have demonstrated fucoxanthin’s robust antioxidant activity, notably its ability to scavenge DPPH radicals, a standard measure of antioxidant capacity. Research using various cell lines such as RAW 264.7, HepG2, Caco-2, and HeLa cells has shown that fucoxanthin reduces cellular metabolic activity and chemiluminescence, indicating a reduction in oxidative reactions, while also elevating intracellular glutathione levels, a critical antioxidant that protects cells from oxidative damage. Fucoxanthin has been shown to inhibit lipid peroxidation, safeguarding cell membranes from oxidative harm [62,63,64].

In vivo studies corroborate these findings, demonstrating that fucoxanthin administration reduces intracellular ROS levels and enhances antioxidant enzyme activity in animal models. For instance, in mice with asthma induced by ovalbumin or liver injury induced by alcohol, fucoxanthin supplementation increased total antioxidant capacity (T-AOC) and levels of SOD, GSH-Px, and catalase, essential for neutralizing ROS and improving organ antioxidant defenses. Activation of the Nrf2 pathway in these studies underscores fucoxanthin’s role in enhancing overall antioxidant defenses [65,66].

**Figure 6:**
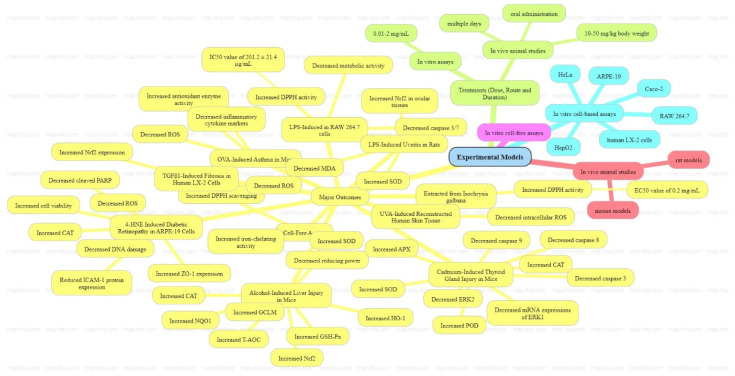
Recent antioxidant activities of fucoxanthin: in vitro and in vivo research. (created with www.map-this.com accessed on 21 May 2024).

Research on human retinal epithelial cells has highlighted fucoxanthin’s protective effects against oxidative stress associated with conditions like diabetic retinopathy, demonstrating increased antioxidant enzyme activity and reduced ROS levels [67,68]. In summary, fucoxanthin and its derivatives exhibit potent antioxidant properties through diverse mechanisms (Figure 6), making them promising candidates for therapeutic applications to mitigate oxidative stress-related pathologies.

### 6.2. Antimicrobial Activities

Fucoxanthin is a promising agent in combating bacterial infections due to its distinct chemical and physical properties and therapeutic potential. Fucoxanthin primarily disrupts several critical processes within bacterial cells, reducing their ability to survive and proliferate. One of the main mechanisms of action of fucoxanthin is its effect of increasing the permeability of the bacterial cell membrane [69]. This change in permeability leads to leakage of cellular contents, similar to the effect observed with specific terpenoids such as carvacrol, thereby enhancing the antimicrobial effects of fucoxanthin. Additionally, research indicates that fucoxanthin disrupts oxidative phosphorylation, a process fundamental to cellular respiration in aerobic bacteria. This disruption reduces intracellular oxygen concentration, making the environment inhospitable for bacteria and promoting their death [70]. At the same time, fucoxanthin promotes the accumulation of intracellular free radicals, causing severe oxidative damage to essential cellular components such as membranes, DNA, proteins, and lipids, thus contributing to its effectiveness against bacteria [71]. Another important mechanism by which fucoxanthin exerts its antibacterial activity is the inhibition of biofilm formation [70]. Bacterial biofilms are aggregates of bacteria surrounded by an extracellular matrix, which makes them highly resistant to antibiotic treatments and the host’s immune defenses. Fucoxanthin disrupts the formation of this biofilm matrix, decreases bacterial cell adhesion, and inhibits toxin production, thereby weakening bacterial virulence. Additionally, fucoxanthin disrupts cellular quorum sensing communication networks, a key mechanism for coordinating bacterial activities within biofilms, further reducing their resilience.

Particularly notable, fucoxanthin demonstrated exceptional activity against *Mycobacterium tuberculosis*, with MICs as low as 2.8–4.1 µM (equivalent to 1.85–2.7 µg/mL) [72]. This bacterium causes tuberculosis, a severe infectious disease affecting millions of people around the world. Additionally, fucoxanthin showed effectiveness against *Listeria monocytogenes* at a concentration of 1000 µg/mL, indicating its potential to combat this potentially deadly food bacteria [73]. Generally, in vitro studies have demonstrated that fucoxanthin has significant activity against various aerobic bacteria. In a study including more than 30 bacterial species, fucoxanthin showed its effectiveness against 13 aerobic bacteria, with MICs generally varying between 62.5 and 250 µg/mL for Gram-positive bacteria and between 125 and 500 µg/mL for Gram-negative bacteria [69]. Table 3 summarizes the main results of studies on the antibacterial activity of fucoxanthin against various bacterial strains, highlighting its potential as an effective antimicrobial agent, particularly against the predominant Gram-positive and Gram-negative pathogens. These results highlight the ability of fucoxanthin to effectively target common bacterial pathogens while mitigating the risk of antibiotic resistance [69].

Additionally, although fucoxanthin has considerable antibacterial activities, its antifungal properties are limited, according to some studies. More research needs to focus on this latter activity. A survey conducted by Pérez et al. (2016) [74] in vitro revealed that different oil extracts of *Saccharina japonica* and *Sargassum horneri*, rich in fucoxanthin, exhibited excellent antifungal properties against *Candida albicans* and *Aspergillus brasiliensis*. According to the results, the mixed acetone–methanol extract of *Sargassum horneri* showed the most potent antimicrobial activity against these two fungi. Maadane et al. (2017) [75] explored some antifungal activities of marine microalgae from the Moroccan coast. Of these, the strain *Phaeodactylum tricornutum*, known for its significant amounts of fucoxanthin, showed moderate results against *Candida albicans*, while *Aspergillus niger* proved resistant. Furthermore, another study by Peraman and Nachimuthu (2019) [76] examined in vitro seven fucoxanthin-containing microalgae (*Pavlova lutheri*, *Isochrysis galbana*, *Navicula* sp., *Chaetoceros calcitrans*, *Dunaliella salina*, *Thalassiosira* sp., and *Chaetoceros gracilis*) for their antifungal activities against *Aspergillus brasiliensis*, *Aspergillus fumigatus*, and *Candida albicans*. At the minimum inhibitory concentration (MIC) of 40 mg/mL, *Dunaliella salina* showed effective antifungal activity against all three fungi, with growth inhibition percentages of 89.26%, 87.67%, and 81.02% for *Aspergillus brasiliensis*, *Aspergillus fumigatus*, and *Candida albicans*, respectively. Notably, *Chaetoceros gracilis* extracts also demonstrated excellent inhibitory activity.

While research on the antiviral properties of fucoxanthin is limited, several studies have introduced new perspectives in pharmacology. For instance, Tsushima et al. (1995) [77] conducted an in vitro study using Raji cells, revealing that fucoxanthin and its metabolites can inhibit Epstein–Barr virus activation induced by 12-O-tetradecanoylphorbol-13-acetate (TPA) at low concentrations. Notably, halocynthiaxanthin, a metabolite derived from fucoxanthin found in marine animals, exhibited significant inhibitory activity, albeit with observed cytotoxicity at higher concentrations [78]. Tamama et al. (2020) [79] explored various fucoxanthin-rich algae capable of modulating the ACE/AngII/ATR1 axis, which could be beneficial in treating patients with COVID-19. However, the precise role of fucoxanthin in this context requires further elucidation. Recent studies by Kang et al. have delved into the antiviral effects of fucoxanthin extracted from *Sargassum siliquastrum*. Kang et al. (2023) [80] demonstrated its ability to inhibit severe acute respiratory syndrome coronavirus 2 (SARS-CoV-2) infection in Vero cells without notable toxicity. Computer simulations suggested that fucoxanthin interferes with the binding between angiotensin-converting enzyme 2 and the virus’s spike protein in a concentration-dependent manner. Moreover, Kang et al. (2024) [81] investigated fucoxanthin’s antiviral activity against the Zika virus in vitro and in silico, highlighting its potential as a promising antiviral agent for emerging viral infections.

### 6.3. Anti-Inflammatory Activities

**Figure 7:**
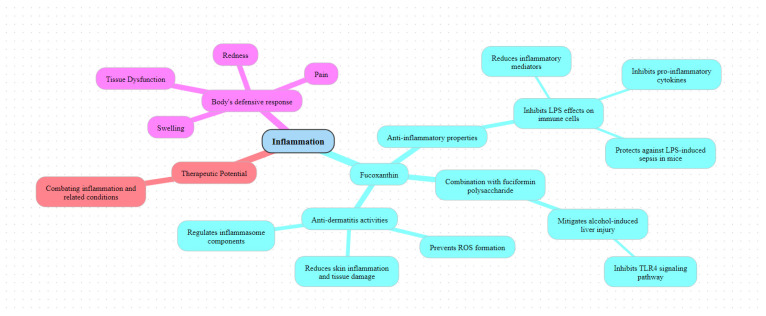
Anti-inflammatory activities of fucoxanthin. (created with www.map-this.com accessed on 22 February 2024).

Inflammation is the immune system’s response to various stimuli, characterized by symptoms such as erythema, edema, pain, and functional changes in tissues (Figure 7). This response can be triggered by different pathogens, including lipopolysaccharide (LPS), a component of the cell walls of Gram-negative bacteria. Fucoxanthin, known for its significant anti-inflammatory properties, is attracting increasing interest and has been extensively studied for its effects on inflammatory processes and potential therapeutic applications [82].

LPS binds to the CD14 receptor, forming immune complexes that interact with Toll-like receptors (TLRs), triggering an intracellular signaling cascade and the release of various inflammatory mediators [83]. Fucoxanthin has demonstrated significant anti-inflammatory and antioxidant effects on LPS-activated murine RAW 264.7 cells. Research shows that fucoxanthin inhibits iNOS expression and COX-2 transcription, reducing NO and PGE2 production. Additionally, it dose-dependently inhibits the stimulation of pro-inflammatory cytokines such as TNF-α and IL-6 and prevents LPS-induced loss of cell viability and mitochondrial membrane potential [84].

Another experimental study demonstrated that fucoxanthin significantly reduced the increased expression of IL-1β, TNF-α, iNOS, and COX-2, attenuating inflammation in LPS-stimulated macrophages [85]. This mechanism involves suppression of IκB-α phosphorylation, MAPKs, and Akt, thereby leading to inactivation of NF-κB and inhibiting LPS-induced secretion of NO and PGE2 by macrophages. These signaling pathways, essential for regulating inflammation and immune responses, play a good role in the anti-inflammatory effects of fucoxanthin.

In vivo studies using mouse models of LPS-induced sepsis confirmed that fucoxanthin decreases the levels of inflammatory cytokines like IL-6 and IL-1β by regulating the NF-κB signaling pathway, thus suggesting its therapeutic potential against sepsis and acute inflammation [86]. Additionally, after excessive alcohol consumption, LPS can activate the TLR4 receptor, thereby amplifying inflammation and leading to liver damage. Administration of fucoxanthin combined with fuciformin polysaccharide significantly inhibited the TLR4 signaling pathway, thereby reducing alcohol-induced liver damage [87].

In addition to its effectiveness against LPS-induced inflammation, fucoxanthin shows promising activities against dermatitis. Combined with rosmarinic acid (RA), it reduces UVB-induced apoptosis of HaCaT keratinocytes by regulating inflammasome components and preventing ROS formation while increasing the expression of antioxidant genes such as Nrf2 and HO-1 [88]. In mouse models, fucoxanthin creams showed significant efficacy against tissue plasminogen activator (TPA)-induced hyperplasia and UVB-induced acute erythema, thereby reducing skin edema, epidermal thickness, MPO activity, and COX-2 expression. Furthermore, fucoxanthin pretreatment improved the symptoms of UVB-induced corneal denervation and trigeminal neuralgia by increasing Nrf2 expression [89].

### 6.4. Anti-Obesity Activity

Obesity has become a major public health issue on a global scale, leading to significant consequences on the metabolic health of individuals (Table 4) [90]. This chronic condition, characterized by excessive accumulation of adipose tissue, is associated with an increased risk of developing several serious metabolic disorders, such as type 2 diabetes, cardiovascular disease, and fatty liver disease. Faced with this growing concern, fucoxanthin and its derivatives are emerging as a promising molecule in the fight against obesity. Several recent studies have highlighted the beneficial effects of fucoxanthin on reducing body fat in obese individuals, sparking growing interest in its therapeutic potential [91].

The mechanisms of action of fucoxanthin against obesity are diverse and complementary. First, fucoxanthin stimulates the expression of Uncoupling Protein 1 (UCP1) in brown adipose tissue (BAT). This protein is essential because it promotes metabolic heat production by dissipating energy, which helps reduce body fat accumulation. Another key mechanism is the browning of white adipose tissue (WAT). Fucoxanthin can transform white adipose tissue deposits into brown adipose tissue, thereby increasing the ability of these cells to oxidize fatty acids and generate heat. This process, known as browning, represents an innovative approach to treating obesity by improving lipid metabolism [92,93].

Furthermore, fucoxanthin and its metabolite, fucoxanthinol, inhibit adipocyte differentiation by negatively regulating gene expression in adipogenesis, such as PPARγ. These compounds also show a significant ability to inhibit pancreatic lipase activity, which reduces the absorption of lipids from the diet and thus limits the accumulation of body fat [94]. Regulation of fatty acid synthesis is another important aspect of fucoxanthin action. By increasing the phosphorylation of AMP-activated protein kinase (AMPK) and inhibiting the activity of acetyl-CoA carboxylase, fucoxanthin helps reduce fatty acid synthesis, thereby improving overall lipid metabolism. Finally, fucoxanthin can inhibit digestive enzymes such as α-amylase and α-glucosidase, which limits the absorption of carbohydrates and contributes to better body weight management in obese individuals. This enzyme inhibition could play an important role in the prevention of type 2 diabetes and other obesity-related metabolic complications [95].

### 6.5. Antidiabetic Activity

Fucoxanthin is gaining attention for its potential therapeutic benefits in managing diabetes, a disease whose global prevalence is projected to increase significantly by 2045 [96]. While diabetes cannot currently be cured, efforts are concentrated on early detection and lifestyle changes to mitigate its complications and mortality rates. Type 2 diabetes pathophysiology is closely linked to obesity, marked by chronic low-grade inflammation and insulin resistance in adipose tissues. Fucoxanthin addresses these issues on multiple fronts. In obesity, hypertrophied adipocytes release proinflammatory cytokines like TNF-α and IL-6, exacerbating systemic inflammation and insulin resistance [97]. Preclinical studies indicate that fucoxanthin can suppress the production of these cytokines, thereby reducing oxidative stress and enhancing insulin sensitivity. By inhibiting MCP-1 production, fucoxanthin also limits macrophage infiltration into adipose tissue, further improving insulin resistance associated with obesity and type 2 diabetes [98].

Fucoxanthin also directly affects carbohydrate metabolism. In animal models fed high-fat diets and diabetic mice (KK-Ay, db/db), fucoxanthin supplementation has shown significant reductions in blood glucose, plasma insulin, and HbA1C compared to controls [99]. These benefits stem from fucoxanthin’s ability to promote GLUT4 translocation in skeletal muscle and adipose tissues, facilitating glucose uptake and utilization. Moreover, fucoxanthin alters adipokine levels in the body. It increases the secretion of adiponectin, an anti-inflammatory adipokine that regulates lipid and glucose metabolism, while decreasing levels of resistin, a pro-inflammatory adipokine linked to insulin resistance and obesity. This modulation helps enhance insulin sensitivity and reduce diabetes-related complications [100].

Fucoxanthin also influences various biological and metabolic parameters critical in diabetes management. It enhances hepatic glucokinase activity, promoting glucose breakdown and glycogen synthesis in the liver to stabilize blood sugar levels and prevent postprandial glucose spikes [101]. Compared to traditional pharmacological treatments like oral hypoglycemic agents and insulin, fucoxanthin offers the advantage of being a natural compound derived from marine algae, potentially offering a safer therapeutic alternative with fewer side effects. Nevertheless, further clinical studies are necessary to assess its efficacy and safety in humans fully.

### 6.6. Other Medical Applications

Fucoxanthin, known for its diverse therapeutic properties, reveals various pharmacological activities beyond its remarkable applications as an anticancer agent, antioxidant, antimicrobial, anti-inflammatory, and other vital effects. These characteristics have prompted extensive research to explore its specific applications in different healthcare fields.

The neuroprotective effects of fucoxanthin have been demonstrated by its ability to safeguard brain cells against oxidative damage induced by agents such as hydrogen peroxide (H_2_O_2_) and β-amyloid oligomers, biomarkers associated with Alzheimer’s disease. Activation of the PI3K/Akt signaling pathway and inhibition of the ERK pathway have reduced inflammation and oxidative stress [102].

Antidepressant properties of fucoxanthin have also been demonstrated, reversing depressive behaviours in animal models exposed to lipopolysaccharide (LPS). This process was mediated by regulation of the AMPK-NF-κB signaling pathway, which decreased the production of pro-inflammatory cytokines and reduced the expression of pro-inflammatory enzymes in the brain [103].

Kidney disease, a major public health issue, has been addressed with the significant renoprotective effects of fucoxanthin, attenuating diabetic nephropathy by reducing oxidative stress and fibrosis via the Akt/Sirt1/FoxO3α signaling pathway. In ethylene glycol-treated albino rats, fucoxanthin normalized biochemical markers and decreased oxidative stress, lipid peroxidation, and tubular damage. In streptozotocin (STZ)-induced diabetic rat models, renal function improvements and mitochondrial integrity protection were observed [104].

Fucoxanthin also favorably influences the lipid profile by increasing the proportion of essential fatty acids like docosahexaenoic acid (DHA) in the liver, thereby reducing the risk of cardiovascular diseases. Protective efficacy against stroke and ischemic neuronal injury was noted, mitigating damage caused by hypoxia and reperfusion [105].

A promising potential of fucoxanthin as an anti-osteoporotic agent has been demonstrated, particularly in postmenopausal women and older people. In ovariectomized (OVX) rat models, bone resorption and inflammatory markers were reduced. Osteoclast differentiation and activity were inhibited via modulation of MAP kinase and Nrf2 signaling pathways [106].

Significant antifibrotic effects of fucoxanthin have been noted in conditions such as pulmonary fibrosis and nasal polyps. A reduction in the expression of proteins such as smooth muscle actin (α-SMA), collagen type I (Col-1), fibronectin, and interleukin-6 (IL-6), induced by TGF-β1, was noted. These effects suppressed phosphorylated MAPK signaling pathways, PI3K/Akt, Akt/SP-1, and Smad2/Smad3 pathways [107].

The anti-hyperuricemic activity of fucoxanthin regulated uric acid transport in the kidneys by modulating the levels of proteins involved in this process, such as GLUT9 and URAT1. This regulation helped lower serum uric acid levels, thereby preventing hyperuricemia, a risk factor for gout and other metabolic complications [108].

The skin, the first barrier against external aggressions, is particularly vulnerable to UV radiation. Fucoxanthin reduced the levels of inflammatory cytokines such as TNF-α and IL-6 via the Nrf2 signaling pathway. In atopic dermatitis models, topical application of fucoxanthin inhibited eosinophil infiltration and IL-33 expression while increasing the expression of regulatory cytokines. ROS production and photodegradation reduction have demonstrated a photoprotective potential against UVA and UVB radiation [109].

## 7. Bioavailability, Stability and Toxicity

The bioavailability of fucoxanthin is essential because it directly influences its effectiveness in the body, depending on factors such as stability, bioaccessibility, and toxicity. Fucoxanthin’s resistance to photolysis, thermodegradation, and oxidation poses a major challenge for its application in medical products and pharmaceutical treatments, potentially compromising its stability and efficacy under adverse environmental conditions. Although fucoxanthin is stable as a free molecule, in vitro digestibility studies show progressive degradation in the gastric simulator (10%) to the ileum (20%), where it is transformed into fucoxanthinol. [89,110] Various strategies are being explored to improve its solubility and stability in the gastrointestinal tract, including encapsulation in lipid nanoparticles, cyclodextrin complexes, and specialized emulsions, such as those based on whey proteins, which are promising in preserving its therapeutic potential.

Concerning toxicity, initial studies indicate that fucoxanthin has low toxicity and good tolerability at therapeutic doses. Research by Beppu et al. (2009) [111] in mice and rats revealed no mortality or histological abnormalities after oral exposure up to 2000 mg/kg/day for 30 days. However, an increase in plasma cholesterol was observed. Subsequent evaluations, including the Ames test and oral toxicity studies, confirmed the absence of mutagenicity, genotoxicity, and toxicity at lethal doses (LD50 > 2000 mg/kg), as well as the absence of abnormalities of internal organs at doses up to 200 mg/kg. Available data suggest that fucoxanthin has a promising safety profile, although continued monitoring is essential for a full assessment of its clinical utility. Further studies will be needed to assess its long-term safety and potential effects at high doses.

To date, although several literature reviews have been published on the various pharmacological properties of fucoxanthin, the distinct pharmacokinetic profile of fucoxanthin at the clinical and preclinical levels is not well documented [112,113,114]. The pharmacokinetics of fucoxanthin aims to show how a substance changes after administration, passing through the phases of absorption, distribution, metabolism, and excretion [109]. In humans, the absorption of fucoxanthin isolated from Laminaria japonica after oral administration has been little studied [115]. Available studies mainly focus on animal models, such as mice, where fucoxanthin has been shown to be rapidly converted to fucoxanthinol and amarouciaxanthin A in blood plasma [116]. One study reported that the metabolites fucoxanthinol and amarouciaxanthin A reach peak plasma concentrations after 4 h, with a peak concentration of fucoxanthinol twice that of amarouciaxanthin A [22]. However, it is essential to note that the mechanisms observed in rodents are not necessarily identical in humans due to significant interspecies differences in carotenoid metabolic pathways. Thus, additional clinical studies are needed to accurately determine the pharmacokinetics of fucoxanthin in humans, particularly with regard to its intestinal absorption and metabolism.

## 8. Challenges and Prospects of Microalgal Fucoxanthin

Fucoxanthin, a prominent carotenoid in certain microalgae, is increasingly recognized for its diverse pharmacological properties. This bioactive compound has garnered attention for its potential as a natural and renewable source, particularly its antioxidant and anticancer effects. However, despite its promising therapeutic potential, fucoxanthin faces several complex challenges that hinder its full utilization in the medical field:

- Efficient Extraction: Extracting fucoxanthin from microalgae efficiently remains a significant hurdle. The carotenoid’s relatively low intracellular concentration necessitates sophisticated and economically viable extraction methods to achieve high yields. Moreover, purifying fucoxanthin to a degree suitable for medical applications requires advanced chromatography and filtration techniques.
- Stability Issues: Fucoxanthin is prone to physicochemical changes under light, heat, and oxygen, compromising its bioavailability and effectiveness in pharmaceutical and nutraceutical formulations. Therefore, developing effective stabilization methods is important to maintain its therapeutic potential.
- High Production Costs: The cost of producing fucoxanthin commercially is prohibitively high. Microalgae cultivation, harvesting, extraction, and purification processes are economically intensive. Reducing these costs while ensuring product quality is essential for broader adoption in medical and nutritional industries.
- Regulatory Standards: Establishing rigorous regulatory standards is essential to ensure fucoxanthin products’ safety, quality, and effectiveness. Harmonized regulations are needed to instill confidence among consumers and healthcare professionals in this promising natural substance.
- Clinical Evaluation: While preclinical studies have demonstrated diverse therapeutic potential, rigorous clinical trials are essential to evaluate fucoxanthin’s efficacy in humans. These studies will determine optimal doses, administration routes, and potential adverse effects associated with medical use.
- Despite these challenges, promising prospects are emerging for fucoxanthin:
- Nutritional and Metabolic Health: Fucoxanthin may contribute to managing body weight and metabolic disorders such as hyperuricemia and hyperlipidemia, suggesting broader applications beyond traditional medicine.
- Formulation Innovations: Innovations in formulation technologies, such as encapsulation in nanoparticles and liposomes, aim to enhance the stability and bioavailability of fucoxanthin, thereby improving its absorption and effectiveness.
- Sustainability: Microalgae, as primary sources of fucoxanthin, offer a sustainable alternative to conventional sources of active compounds. Their controlled cultivation reduces environmental impact compared to agricultural or synthetic methods, supporting sustainable production practices.
- Education and Awareness: Educating consumers and healthcare professionals about the potential benefits and proper use of fucoxanthin is essential for widespread adoption. This includes raising awareness about quality challenges and regulatory considerations associated with products containing this carotenoid.

In summary, while fucoxanthin faces significant hurdles, ongoing research and innovation hold promise for unlocking its full potential in both therapeutic and nutritional applications.

## 9. Conclusions

Fucoxanthin, a bioactive carotenoid found abundantly in microalgae, has garnered significant attention for its diverse therapeutic potential across various medical fields. Its promising anticancer properties are particularly intriguing to researchers striving to advance innovative therapies for human health improvement. Beyond its anticancer effects, fucoxanthin exhibits many beneficial properties, including antioxidant, antimicrobial, anti-inflammatory, anti-obesity, antidiabetic, neuroprotective, and antidepressant activities. Current medical research is focused on elucidating the mechanisms through which fucoxanthin operates and exploring its synergistic interactions with other bioactive compounds. These efforts aim to optimize its beneficial effects in stable pharmaceutical formulations, facilitating precise and practical clinical applications. The functional versatility of fucoxanthin, combined with its abundant presence in microalgae biomass, positions it as a renewable and potentially sustainable source of this valuable compound. This aspect holds promise for developing innovative and enduring therapeutic solutions within modern medical technology.

## Figures and Tables

**Table 2:** Main anticancer activities of fucoxanthin.

Organs	Mechanisms of Action
Brain	Induction of apoptosis of glioma cells. Inhibition of the PI3K/Akt/mTOR pathway.
Breasts	Inhibition of tumor angiogenesis. Reduced viability of breast cancer cells. Protection against doxorubicin cardiotoxicity.
Lungs	Prevention of the development of non-small cell lung cancer. Induction of apoptosis by regulation of the expressions of p53, p21, Fas, PUMA, Bcl-2, and caspase-3/8. Cell cycle inhibition and reduction of mortalin–p53 interaction. Reduction of proteins linked to proliferation, survival, and metastasis of cancer cells.
Cervix	Synergy with TRAIL to increase apoptosis. Targeting the PI3K/Akt/NF-κB pathway.
Colon	Induction of DNA fragmentation. Inhibition of tumor growth.
Liver	Protection of hepatocytes. Reduction of lipid peroxide metabolites. Reduction of stress markers.
Blood	Cytotoxicity for leukemia cells. Induction of apoptosis by mitochondrial and caspase-3 pathways.
Stomach	Reduction in the expression of Mcl-1 and STAT3 proteins. Induction of apoptosis and autophagy.

**Table 3:** Main results of the antibacterial activities of fucoxanthin.

Bacterial Strain	ZOI * (mm)	MIC ** (µg/mL)
*Streptococcus agalactiae*	12.2 ± 0.7	62.5
*Staphylococcus epidermidis*	11.2 ± 0.7	125
*Staphylococcus aureus*	11.0 ± 0.6	125
*Escherichia coli*	10.2 ± 0.7	125
*Streptococcus pyogenes*	10.0 ± 0.6	125
*Klebsiella oxytoca*	9.2 ± 0.7	125–250
*Enterococcus faecalis*	9.0 ± 0.8	125–250
*Streptococcus pneumoniae*	9.7 ± 0.5	125
*Staphylococcus aureus*	11.0 ± 0.6	125
*Klebsiella pneumoniae*	8.8 ± 0.7	250
*Acinetobacter lwoffii*	8.2 ± 0.4	250
*Pseudomonas aeruginosa*	7.5 ± 0.5	250–500
*Serratia marcescens*	7.3 ± 0.5	500
*Proteus mirabilis*	7.2 ± 0.4	500
*Cutibacterium acnes*	6.0 ± 0.1	>1000
*Veillonella parvula*	6.0 ± 0.1	>1000
*Porphyromonas gingivalis*	6.0 ± 0.1	>1000

* ZOI: zone of growth inhibition. ** MIC: minimal inhibitory concentration.

**Table 4:** Mechanisms of action of fucoxanthin against obesity.

Mechanism of Action Enzymatic	Description	Reactions Involved
Regulation of UCP1	Stimulates the expression of Uncoupling Protein 1 (UCP1) in brown adipose tissue (BAT).	Unspecified
Browning of white adipose tissue (WAT)	Transforms some white adipose tissue depots into brown adipose tissue via induction of UCP1.	Unspecified
Inhibition of adipogenesis	Inhibits adipocyte differentiation by negatively regulating adipogenic genes like PPARγ.	PPARγ inhibition Pancreatic lipase inhibition
Reduced fatty acid synthesis	Increases phosphorylation of AMP-activated protein kinase (AMPK).	Reduced acetyl-CoA carboxylase activity
Inhibition of digestive enzymes	Inhibits the enzymes α-amylase and α-glucosidase, reducing glucose absorption.	Inhibition of α-amylase Inhibition of α-glucosidase
